# Can we predict long-term survival in resectable pancreatic ductal adenocarcinoma?

**DOI:** 10.18632/oncotarget.26511

**Published:** 2019-01-22

**Authors:** Tamara M.H. Gall, Gareth Gerrard, Adam E. Frampton, Leandro Castellano, Raida Ahmad, Nagy Habib, Duncan Spalding, Madhava Pai, Letizia Foroni, Long R. Jiao

**Affiliations:** ^1^ Department of Surgery and Cancer, Imperial College, Hammersmith Hospital Campus, London W12 0HS, United Kingdom; ^2^ Faculty of Medicine, Imperial College, Hammersmith Hospital Campus, London W12 0HS, United Kingdom; ^3^ Current address: Sarah Cannon Molecular Diagnostics, HCA Healthcare UK, London WC1E 6JA, United Kingdom; ^4^ Department of Histopathology, Imperial College, Hammersmith Hospital Campus, London W12 0HS, United Kingdom

**Keywords:** PDAC, ion torrent, next-generation sequencing, nanostring, cell-free DNA

## Abstract

**Objective:**

Pancreatic ductal adenocarcinoma (PDAC) is an aggressive tumour associated with poor 5-year survival. We aimed to determine factors which differentiate short and long-term survivors and identify a prognostic biomarker.

**Methods:**

Over a ten-year period, patients with resected PDAC who developed disease recurrence within 12 months (Group I) and those who had no disease recurrence for 24 months (Group II) were identified. Clinicopathological data was analysed. Ion Torrent high-throughput sequencing on DNA extracted from FFPE tumour samples was used to identify mutations. Additionally, peripheral blood samples were analysed for variants in cell-free DNA, circulating tumour cells (CTCs), and microRNAs.

**Results:**

Multivariable analysis of clinicopathological factors showed that a positive medial resection margin was significantly associated with short disease-free survival (*p* = 0.007). Group I patients (*n* = 21) had a higher frequency of the *KRAS* mutant mean variant allele (16.93% ± 11.04) compared to those in Group II (*n* = 13; 7.55% ± 5.76, *p* = 0.0078). Group I patients also trended towards having a *KRAS* c.35G>A p.Gly12Asp mutation in addition to variants in other genes, such as *TP53*, *CDKN2A*, and *SMAD4*. Mutational status of cell-free DNA, and number of CTCs, was not found to be useful in this study. A circulating miRNA (hsa-miR-548ah-5p) was found to be significantly differentially expressed.

**Conclusions:**

Medial resection margin status and the frequency of *KRAS* mutation in the tumour tissue are independent prognostic indicators for resectable PDAC. Circulating miRNA hsa-miR-548ah-5p has the potential to be used as a prognostic biomarker.

## INTRODUCTION

Pancreatic ductal adenocarcinoma (PDAC) is an aggressive disease with an extremely poor prognosis. The GLOBOCAN project estimated that worldwide there are 337,872 new cases of PDAC diagnosed each year [1]. Although this only accounts for 3% of new cancer cases, it is the fourth leading cause of cancer death. Surgical resection is the only potentially curative treatment; however, even those with an R0 resection have a median survival of just 29 months [2]. Determining factors that lead to disease recurrence may help identify those with poor prognosis to allow personalised treatment options. Indeed, recent results from the Know Your Tumour Initiative, showed that survival is improved when matched therapy is given to patients with highly actionable proteomic alterations [3].

In 2008, an extensive land-mark study characterised the genetic network of PDAC [4]. It was established that four genes are most commonly affected in PDAC; predominately activating mutations in *KRAS* driving transformation, with concomitant loss-of-function mutations in *CDKN2A, TP53* and *SMAD4* associated with progression. There are a few published reports that *KRAS* mutational status is associated with shorter survival in PDAC patients [5, 6] Further, there is interest in developing prognostic biomarkers for PDAC, with the mutational status of cell-free DNA, the number of circulating tumour cells, and the expression of circulating microRNAs being of current interest to pancreatic cancer research [7]. We therefore aimed to establish whether any clinicopathological factors or genetic mutational differences existed between short and long disease-free survivors following surgical resection of PDAC. Further, we sought to identify a prognostic biomarker.

## RESULTS

### Clinicopathological factors

Over a 10-year period, 654 patients had pancreatic resections. Of these, 178 were for a histopathological diagnosis of PDAC, with 168 undergoing curative resections. The remaining 10 patients had locally advanced disease or metastatic disease evident intra-operatively. A further 16 patients had an R2 resection and were excluded from the analysis. Of the remaining 152 patients, 68 (44.74%) had evidence of disease recurrence within 12 months of surgery (Group I) and 25 (16.45%) had no evidence of disease recurrence for at least 24 months after surgery (Group II). These 93 patients were included in the analysis. Clinicopathological characteristics of the patients and tumours are shown in Table 1. There was no 30-day mortality. Complications occurred in 30.61% with Clavien-Dindo classification 3a-4 complication [8]. occurring in 6 patients (6.45%), four required radiological intervention for post-operative intra-abdominal collections and 2 (2.15%) required further operations for bleeding from the pancreatic stump.

**Table 1:** Clinicopathological characteristics of patients in Group I (early recurrence) and Group II (late recurrence) and *p*-value results of univariate analysis

Characteristic	All patients (*n* = 93)	Group I (*n* = 68)	Group II (*n* = 25)	*p* value
Median age years (range)	66.66 (40.71–82.82)	66.18 (40.71–82.82)	67.81 (49.63–75.36)	0.697
Sex	52:41	35:33	17:8	0.168
M:F (%)	(56:44)	(51:49)	(68:32)	
Median preoperative	87.00	73.00	198.00	0.347
CA 19-9	(1–32,876)	(1–32,876)	(1–10,195)	
Kunits/L, (range)				
(*n* = 54)				
Preoperative CA 19-9	37:17	28:9	9:8	0.121
<37:>37 (%)	(69:31)	(76:24)	(53:47)	
(*n* = 54)				
Median preoperative bilirubin umol/L (range)	18	18	17	0.877
(*n* = 89)	(2–494)	(3–248)	(2–494)	
Preoperative bilirubin	52:37	39:27	13:10	1.000
<22:>22 (%)	(58:42)	(59:41)	(57:43)	
(*n* = 89)				
Median preoperative CRP mg/L (range)	6	6	6	0.854
(*n* = 89)	(1–141)	(1–141)	(1–141)	
Preoperative CRP	66:23	48:18	18:5	0.783
<20:>20 (*n* = 89)	(74:26)	(73:27)	(78:22)	
Type of operation	81:12	58:10	23:2	0.503
PD:DP (%)	(87:13)	(85:15)	(92:8)	
Morbidity no:yes (%)	15:34	11:24	4:10	1.000
(*n* = 49)	(31:69)	(31:69)	(29:71)	
Differentiation poor:moderate-to-poor:moderate:well (%)	55:10:27:1	40:9:18:1	15:1:9:0	0.495
	(59: 11: 29:1)	(59: 13: 26: 1)	(60: 4: 36: 0)	
Resection margin status	45: 48	25:43	20:5	**0.000**
R0:R1 (%)	(48:52)	(37: 63)	(80:20)	
Medial resection margin status	57:36	33:35	24:1	**0.000**
R0:R1 (%)	(61:39)	(49:51)	(96:4)	
Anterior resection margin status	81:12	59:9	22:3	1.00
R0:R1 (%)	(87:13)	(87:13)	(88:12)	
Posterior resection margin status	81:12	56:12	25:0	**0.032**
R0:R1 (%)	(87:13)	(82:18)	(100:0)	
Pancreatic resection margin status	86:7	65:3	21:4	0.081
R0:R1 (%)	(92:8)	(96:4)	(84:16)	
Median tumour size cm (range)	2.8	3.0	2.2	**0.002**
	(0.8–6.5)	(0.8–6.5)	(0.9–4.5)	
T-stage	3:8:63:19	3:2:47:16	0:6:16:3	**0.014**
1:2:3:4 (%)	(3:9:68:20)	(4:3:69:24)	(0:24:64:12)	
N-stage	22:71	10:58	12:13	**0.002**
N0:N1 (%)	(24:76)	(15:85)	(48:52)	
Lymph node ratio	21:29	18:24	3:5	1.000
<0.2: >0.2 (%)	(42:58)	(43:57)	(38:63)	
(*n* = 50)				
Perineural invasion absent:present (%)	20:73	13:55	7:18	0.398
	(22:78)	(19:81)	(28:72)	
Lymphovascular invasion absent:present (%)	21:72	11:57	10:15	**0.018**
	(23:77)	(16:84)	(40:60)0.018	
Adjuvant chemotherapy no:yes (%)	14:79	12:56	2:23	0.338
	(15:85)	(18:82)	(8:92)	
Mortality no:yes (%)	14:79	3:65	11:14	**0.000**
	(15:85)	(4:96)	(44:56)	

Comparisons were made using Fisher’s exact test for categorical data and the Wilcoxon signed rank test for continuous data. All tests were two-sided.

On univariate analysis, factors associated with short disease-free survival compared to long disease-free survival were: resection margin status (*p* < 0.001); medial resection margin status (*p* < 0.001), posterior resection margin status (*p* = 0.032), tumour size (*p* = 0.002); T-stage (*p* = 0.014); N-stage (*p* = 0.002); and the presence of lymphovascular invasion (*p* = 0.018) (Table 1). On multiple regression analysis, including these factors which significantly differed between the two groups, only medial resection margin status was independently associated with short disease-free survival (*p* = 0.007, OR 0.028, 95% CI 0.002–0.383) (Table 2).

**Table 2:** Multivariate analysis of clinicopathological factors that were significantly different between each group on univariate analysis

Characteristic	*p*-value univariate analysis	*p*-value multivariate analysis	OR	95% CI for OR
Resection margin status	0.000	0.677	1.444	0.256–8.125
Medial resection margin status	0.000	**0.007**	**0.028**	**0.002–0.383**
Posterior resection margin status	0.032	0.998	0.000	0.000
Tumour size	0.002	0.132	0.593	0.301–1.170
T-stage	0.014	0.556	1.324	0.520–3.372
N-stage	0.002	0.124	0.370	0.104–1.313
Lymphovascular invasion	0.018	0.229	0.418	0.101–1.734

Abbreviations: OR = Odds ratio. CI = Confidence interval.

No patients included in this analysis were lost to follow-up following resection. Mean follow-up to date of death or to final follow-up for patients still alive was 24.8 months (range 1.5–108.1). By definition all the short disease-free survival group had disease recurrence. Nine (36%) of the long disease-free survival group, developed disease recurrence after 24 months. Median overall survival was 9.8 months (95% CI 9.0–10.6) for Group I (short disease-free survival) which was significantly less than the 56.1 months (95% CI 34.4–77.8) for Group II (long disease-free survival; *p* < 0.001).

### Ion torrent sequencing

#### Technical performance of the ion ampliseq cancer hotspot panel v2

DNA was extracted from macrodissection of tumour FFPE blocks in 34 patients. All samples (Group I *n* = 21; Group II *n* = 13) were processed once with an 81% success rate. Samples with poor sequence reads (amplicon drop-out or mean coverage <100 reads) were processed a second time. Successful results were generated in 18 patients from Group I and 11 patients from Group II. Mutations in 34 loci of 12 genes were identified as well as 382 single nucleotide polymorphisms (SNPs). The mean read length was 103bp with a mean of 197,452 reads per sample.

#### KRAS mutations

**Figure 1:**
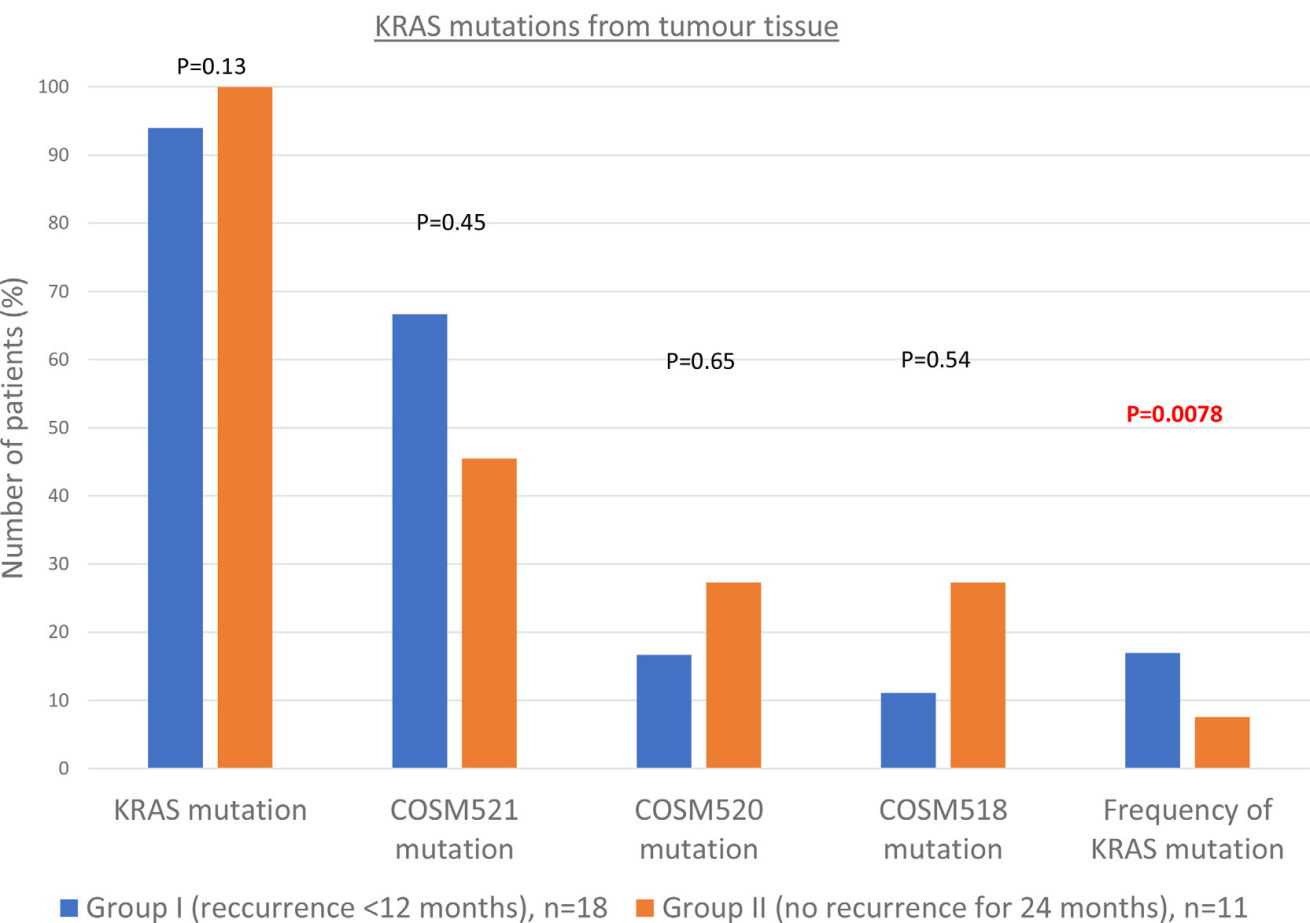
Results of next generation DNA sequencing Shows the percentage of each type of *KRAS* gene mutation for Group I (early recurrence) and Group II (late recurrence).

17/18 (94%) of group I samples contained a KRAS point mutation. Of these: 70.59% were c.35G>A p.(Gly12Asp) (COSM521) and 17.65% were c.35G>T p.(Gly12Val) (COSM520). The mean frequency of the mutation found in each sample was 16.93 ± 11.04%. This compares to 11/11 (100%) of group II samples, which contained a KRAS point mutation (*p* = 0.13). These were COSM521 in 45.45% (*p* = 0.45), COSM520 in 27.27% (*p* = 0.65) and c.34G>C p.(Gly12Arg) (COSM518) in 27.27% (*p* = 0.54). The mean frequency of the mutation found in each sample was 7.55 ± 5.76%, *p* = 0.0078. See Figure 1.

*TP53, CDKNA2, SMAD4* and multiple *mutations:* Results summarized in Table 3. Interestingly, 61% of early recurrence patients compared to 27% of late recurrence patients (*p* = 0.12) had a *KRAS* mutation and a mutation in either *TP53, CDKN2A* or SMAD4. Of note, we are aware that some alterations in *CDKN2A* may be missed due to large deletions not being picked up by the iontorrent method.

**Table 3:** Results of next generation DNA sequencing

Gene mutation	Group I, *n* = 18	Group II, *n* = 11	*p* value
*TP53*	8 (44%)	3 (27%)	0.86
*CDKN2A*	2 (11%)	0 (0%)	0.25
*SMAD4*	2 (11%)	0 (0%)	0.25
2 or more mutations	11 (61%)	5 (48%)	0.41
*KRAS* + *TP53*, *CDKN2A* or *SMAD4*	11 (61%)	3 (27%)	0.12

Shows the number of *TP53*, *CDKN2A*, *SMAD4* and multiple mutations for Group I (early recurrence) and Group II (late recurrence).

On regression analysis, both high frequency of *KRAS* mutation and a positive medial resection margin, retain their significance (*p* = 0.002 and *p* = 0.022 respectively).

### Peripheral blood results

#### cfDNA sequencing

Plasma samples from the peripheral circulation were collected from a total of sixteen patients (8 in Group I and 8 in Group II). A mean of 63.67 ± 24.37 ng/μl of DNA was extracted from each sample. A genetic mutation was identified in two samples: an APC gene mutation and an *STK11* mutation, the location of which corresponded to the tumour sample mutations seen in these patients. No other mutations, specifically no *KRAS* mutations were identified in any samples.

#### Circulating tumour cells (CTCs) analysis

CTC analysis was undertaken from the peripheral circulation blood samples in sixteen patients (8 in Group I and 8 in Group II). Four patients (50%) in Group I and 4 patients (50%) in Group II had at least 1 CTC detected. No more than 2 CTCs were seen in any sample.

#### microRNA nanoString analysis

Blood from the sixteen patients used above was also analysed for miRNAs. The five miRNAs which were the most differentially expressed between patients with short disease-free survival and those with long disease-free survival were: hsa-miR-548ah-5p; hsa-miR-550b-3p; hsa-miR-223-3p; hsa-let-7b-5p and hsa-let-7c. Three were downregulated and two upregulated in Group I compared to Group II. Only one of these miRNAs (hsa-miR-548ah-5p) was significantly differentially expressed between the two groups (Table 4). These miRNAs were entered into the MiRTarBase database. One of these differentially expressed miRNAs is known to target genes identified as mutated in the genetic sequencing of our PDAC samples, namely hsa-let-7b-5p (target KRAS). None of these miRNAs have been previously identified as prognostic indicators in pancreatic cancer.

**Table 4:** The five most differentially expressed microRNAs between Group I and Group II

miRNA	Base mean	Log2 fold change	Adjusted *p*-value	Upregulated/downregulated in Group I compared to Group II	PDAC gene target
hsa-miR-548ah-5p	42.63822508	–0.712787648	**0.001728**	Down-regulated	Nil known
hsa-miR-550b-3p	46.53592309	–0.61247248	**0.245326**	Down-regulated	Nil known
hsa-miR-223-3p	155.6722423	0.603150078	**0.080409**	Up-regulated	Nil known
hsa-let-7b-5p	60.40780961	0.607372968	**0.085134**	Up-regulated	**KRAS**
hsa-let-7c	41.9846227	–0.346058076	**0.082915**	Down-regulated	Nil known

## DISCUSSION

Over a ten-year period, our centre performed 152 curative resections for pancreatic ductal adenocarcinoma. Forty-five per cent developed disease recurrence within 12 months and 16% did not develop disease recurrence for at least two years. We investigated: clinicopathological factors; tumour DNA; and peripheral blood samples for cell-free DNA, circulating tumour cells and microRNAs; in order to identify predictors of long-term survival.

Although a number of clinicopathological factors were found to be associated with early recurrence on univariate analysis, only a positive medial resection margin was associated with early disease recurrence on multivariate analysis. This is consistent with several other retrospective trials which have previously identified resection margin status as an independent prognostic factor in PDAC patients [9–14]. In the European Study Group for Pancreatic Cancer-1 (ESPAC-1) study, those with an R1 resection status had a median survival of 10.9 months versus 16.9 months for patients with R0 margins [15].

We performed high-throughput DNA sequencing using the Ion Torrent multigene next generation sequencer on FFPE tumour samples. We identified that tumour samples from those with early recurrence had a higher frequency of *KRAS* mutation than from those with late recurrence. A higher frequency of mutant *KRAS* may be related to increased tumour density, although this was not evaluated in the current study. Indeed, improved survival is seen in PDAC patients with tumour infiltrating lymphocytes [16] and, as lymphocytes may cause pancreatic tumour cell apoptosis, increased tumour density may reflect an absence of tumour infiltrating immune cells.

*KRAS* mutations were detected in 94% of the tumour samples from Group I (early recurrence) and 100% of tumour samples from Group II (late recurrence). This is repeatedly reported as the most commonly mutated gene in PDAC and, in agreement with others [17], we did not find that mutated *KRAS* was a prognostic factor. However, notably, the long disease-free survivors had fewer patients with a COSM521 mutation and more patients with a COSM518. Although this result did not reach significance, it suggests that the type of *KRAS* mutation may have different effects on tumourigenesis. Indeed, an analysis of 27 PDAC tumour samples also suggested that those with a COSM521 mutation had worse survival than those with other *KRAS* mutations [18].

We identified *TP53* mutations in 44% of tumour samples from the short disease-free survivors and 27% of those from the long disease-free survivors. This is in concordance with the literature where *TP53* is repeatedly shown to be the second most commonly mutated gene in PDAC. Although the difference in mutational status between our two groups did not reach significance, it indicates a trend that patients with *TP53* mutations have worse survival. Indeed, mutant *TP53* has been associated with an increased chemoresistance to gemcitabine [19].

Considering each driver mutation in PDAC acts through differing pathways, we would expect multiple mutations to worsen tumourigenesis and link to survival. Particularly when the activating *KRAS* mutation, is in combination with a tumour suppressor gene. Certainly, when mouse models with activating *KRAS* mutations are combined with a loss-of-function *TP53* and/or *CDKN2A* mutation, an acceleration of PDAC growth, more genetic instability and a tendency for poorly differentiated tumours is seen [20]. Although we found no significant differences between our two groups in terms of number of diver mutations identified, there was a trend towards more mutations in the short disease-free survival group. This is in agreement with others who have found worse survival in patients with concomitant *KRAS* and *CDKN2A* mutations [21], and the number of driver gene alterations has been found to be an independent prognostic factor for overall survival [22].

The Ion Torrent PGM sequencer has been used by others to detect mutations in cfDNA of patients with other tumour types [23–25] Genetic sequencing of blood DNA in our study did not reveal any mutations in the four driver genes associated with PDAC despite the Ion Torrent performing technically well. Interestingly the two gene variants were germline in *STK11* and in *APC*, suggesting that these mutation defects may have been causative rather than resultant. Other studies have detected *KRAS* mutations in the blood of 33–54.5% of PDAC patients [26–31]. However, the majority of these investigated those with unresectable disease and thus at a later stage than our patient cohort. Patients who have resectable pancreatic cancer may have not yet shed tumour cells with DNA containing the mutational defect into the systemic circulation.

There were also no differences seen in the number of CTCs detected in the peripheral circulation between the early and late recurrence groups. We found CTCs in 50% of samples, similar to the literature, where CTCs have been detected in the blood of 43–80% of PDAC patients [32–34]. A shorter progression-free survival has previously been correlated with the detection of CTCs, however most of these studies were conducted in patients with unresectable disease. In a study conducted on patients with resectable PDAC, only 25% had detectable CTCs and, in agreement with our study, these patients did not have shorter disease-free survival [35]. Although the CellSearch system is the most commonly used for CTC detection, it may be that PDAC cells are not captured by this method. Indeed, it assumes that cancer cells have epithelial tissue by relying on the detection of cytokeratins 8- 18- and 19-. However, some cancer cells undergo an epithelial-mesenchymal-transition (EMT), resulting in the downregulation of EpCAM and some cytokeratins [36]. EMT facilitates cancer invasion, so the known aggressive nature of PDAC would suggest that many of its cells undergo an early EMT. Newer technologies for CTC detection, including the ISET system and analysis of CTCs expressing tumour-initiating cell phenotype markers, has been shown to predict survival [37].

We identified five circulating miRNAs that were the most differentially expressed between patients with short- and those with long- disease free survivals. One of these was significantly differentially expressed (hsa-miR-548ah-5p). Although none of these miRNAs have been previously reported as being of prognostic significance in PDAC, the let-7 family is known to target *KRAS*.

Lethal-7 (let-7) is able to regulate *RAS* expression [38]. In cancer, members of the let-7 family are widely reported as tumour suppressor miRNAs with down-regulation observed in many cancer types [39]. Reduced let-7 expression corresponds to elevated *RAS* expression [40] and increased let-7 expression suppresses RAS and abolishes tumorigenesis [41, 42]. In PDAC, let-7 is up-regulated in normal ductal cells compared to PDAC cell-lines [43]. In cells with mutated *KRAS*, inhibition of cell proliferation was seen with the introduction of let-7a [43]. In human patients, Ali *et al.* [44], found that let-c and d were down-regulated in PDAC samples compared to samples from the normal pancreas. This supports our finding of down-regulated let-7c in the short disease-free survival group and a tumour suppressor role of let-7c in PDAC. In contrast, Henry *et al.* [45] found let-7b to be up-regulated in the aspirate of cancerous cystic tumours, which supports our finding of up-regulated let-7b in the short disease-free survival group and an oncogenic role of let-7b in PDAC.

This was a limited study in terms of sample size and therefore subject to the effect small sample sizes have on extreme outcomes, putting our data at risk of a type II error. Further, PDACs exhibit tumour mutational heterogeneity and our tumour DNA was only extracted from one part of each patient’s tumour.

## MATERIALS AND METHODS

### Ethics

This study was approved by a UK national research ethics committee (London; 09/H0722/77) and by our institutional review board (Hammersmith Hospital, Imperial Healthcare NHS Trust). Informed written patient consent for research was obtained before surgical treatment.

### Patients

Patients who had a pancreatic resection over a ten-year period were included in our retrospectively created database. Inclusion factors were: patients with histopathologically confirmed pancreatic ductal adenocarcinoma (PDAC); patients who developed disease recurrence (either local or distant disease) within 12 months from surgery (Group I) or who had no disease recurrence for at least 24 months (Group II). A disease free interval of 12 months has been objectively determined to define early recurrence for resected PDAC [46]. Considering the CONKO-001 study [47] estimated that less than a quarter of resected PDAC patients survive 2 years, we used 24 months as an arbitrary cut-off for long-term disease free survival. Exclusion factors included: patients with abandoned resections (due to locally advanced disease or metastases seen intra-operatively); or R2 resections. Surgical decision was made at our hepatobiliary multidisciplinary team meeting. Surgery was offered to those with: absence of extrapancreatic disease; no involvement of the superior mesenteric artery (SMA); no encasement of the superior mesenteric vein (SMV) or portal vein (PV) >180°; and fit to undergo major surgery (WHO performance status of 0–2). For patients with local venous invasion with <180° encasement, resection and reconstruction of the SMV or PV was performed as required.

### Operative procedures

All operations in our unit were performed by consultant hepatopancreatobiliary surgeons and have been previously described [48]. Both Whipple procedure and distal pancreatectomy with splenectomy are routinely performed either laparoscopically or as an open procedure. The pancreatico-enteric anastamosis was decided during the procedure by the surgeon and performed as either a pancreaticojejunostomy or pancreaticogastrostomy.

### Histopathological examination

The examination of specimens was performed by a hepatopancreaticobiliary histopathologist according to the guidelines of the Royal College of Pathologists. Microscopic evidence of tumour involvement within 1 mm of the margin resulted in an R1 classification [49].

### Follow-up

All patients had routine follow-up in the outpatient clinic at 3 monthly intervals for the first year and then six monthly intervals. Clinical examination, routine laboratory tests and CT scan was organised at each appointment. Date of disease recurrence was recorded as the date of the first CT scan to diagnose abnormal pathology. Date of death was determined from hospital and GP records. Disease-free and overall survival was defined as the interval between date of surgery and date of death or final follow-up.

### DNA high-throughput panel sequencing

#### Tumour samples

The formalin-fixed paraffin-embedded (FFPE) blocks and corresponding H&E stained slides for patients in Group I and Group II were collected. These were reviewed with a specialist pancreatic pathologist (RA) and areas of PDAC were marked. FFPE blocks corresponding to each H&E slide were cut. Macrodissection of the tumour cells was then conducted.

#### DNA extraction from microdissected FFPE blocks

The QIAamp DNA FFPE kit (Qiagen, Hilden, Germany) was used to extract genomic DNA from the FFPE tissues as per the manufacturer’s instructions. DNA quantification of each sample was established with the Qubit 2.0 fluorometer (ThermoFisher, Massachusetts, USA) and the samples were stored at 4° C.

#### Ion torrent sequencing

The Ion Torrent Personal Genome Machine (PGM) next generation sequencer (Life Technologies, Carlsbad, USA) [24, 50], was used to sequence the DNA for each sample according to the manufacturer’s protocols. 10 ng of DNA for each sample was used for library preparation with the Ion AmpliSeq Cancer Hotspot Panel v2 (Life Technologies) which covers 2,800 COSMIC notated mutations from 50 genes, including *KRAS*, *CDKN2A*, *SMAD4* and *TP53.* Data analysis was carried out with Torrent Suite Software (Life Technologies).

#### Blood samples

Patients were invited to donate blood prior to their pancreatic resection. Blood was taken and processed as follows.

#### For DNA sequencing

An EDTA tube of whole blood was collected and DNA was extracted from the plasma using the QIAamp DNA Blood Mini Kit (Qiagen) according to the manufacturer’s protocol. DNA quantification of each sample was established with the Qubit 2.0 fluorometer and the samples were stored at –20° C. Sequencing of DNA was carried out with the ion torrent next generation sequencer as described above.

#### For circulating tumour cells (CTCs) analysis

7.5 mls of whole blood was collected in a CellSave (Veridex, NJ, USA) preservative tube. The samples were then processed in our laboratory within 48 hours using the bead-based fluorescence CellSearch system (Veridex, NJ, USA) according to the manufacturer’s protocol. Samples were then scanned on the CellTracks analyzer II fluorescent microscope (Veridex, NJ, USA). The cells were evaluated for CTCs independently by 2 operators.

#### microRNA extraction

Blood was collected into two EDTA tubes and immediately put on ice. After centrifugation, plasma was stored at −80° C. RNA extraction was performed at a later date using the miRNeasy serum/plasma kit (Qiagen) according to the maufacturer’s protocol. RNA quantification was established with the NanoDrop 2000c UV-Vis spectrophotometer (ThermoFisher).

#### microRNA analysis

miRNA analysis of the extracted RNA was performed using the NanoString nCounter system (NanoString Technologies, Seattle, WA). The method measures a total of 800 probes. miRNA data analysis was performed using the nSolver software analysis, (nanoString Technologies, Seattle, USA). The serum miRNA profiling data was normalized using the average signals obtained from a spike-in control (Lyophilized C. elegans miR-39 miRNA mimic, Qiagen), and miRNAs that gave significant hybridization signals were used for downstream analysis.

#### Statistical analysis

The statistical analyses were performed using IBM SPSS statistics version 21 software package (IBM SPSS, Chicago, USA). The two groups consisted of those with disease recurrence within 12 months and those with no recurrence for at least 24 months. Univariate analysis comparisons were made using Fisher’s exact test/Chi-squared test for categorical data and the mann whitney/ *t*-test - for continuous data. All tests were two-sided. Variables that were significantly different (*p* < 0.05) were included in a multiple regression analysis to examine the impact of these variables on early disease recurrence. Overall survival rates were calculated by Kaplan–Meier method and compared by the log-rank test.

## CONCLUSIONS

A positive medial resection margin and a higher frequency of KRAS mutation in tumour tissue are independent prognostic indicators for resectable PDAC. Further, short disease-free survivors trended towards having a COSM521 mutation of *KRAS* and multiple mutations, particularly of *p53*, *CKDKN2A,* and *SMAD4*. Mutational status of cell-free DNA and CTCs were not found to be useful blood-based biomarkers in PDAC. However, the expression of a circulating microRNA - hsa-miR-548ah-5p may differentiate between short and long-term survivors, which could lead to a more tailored and personalised management approach.
